# Organically modified layered magnesium silicates to improve rheology of reservoir drilling fluids

**DOI:** 10.1038/s41598-020-70752-1

**Published:** 2020-08-17

**Authors:** Hasmukh A. Patel, Ashok Santra

**Affiliations:** Drilling Technology Team, Aramco Americas: Aramco Research Center – Houston, 16300 Park Row Dr, Houston, TX 77084 USA

**Keywords:** Inorganic chemistry, Materials chemistry, Physical chemistry, Surface chemistry

## Abstract

Petroleum well drilling fluids are one of the most significant constituents in the subterranean drilling processes to meet an increasing global demand for oil and gas. Drilling fluids experience exceptional wellbore conditions, e.g. high temperature and high pressure that adversely affect the rheology of these fluids. Gas and oil well drilling operations have to adjourn due to changes in fluid rheology, since the drilling fluids may lose their effectiveness to suspend heavy particles and to carry drilled cuttings to the surface. The rheological properties of drilling fluids can be controlled by employing viscosifiers that should have exceptional stability in downhole environments. Here, we have developed next-generation viscosifiers—organically modified magnesium silicates (MSils)—for reservoir drilling fluids where organic functionalities are directly linked through the Si–C bond, unlike the industry’s traditional viscosifier, organoclay, that has electrostatic linkages. The successful formation of covalently-linked hexadecyl and phenyl functionalized magnesium silicates (MSil-C16 and MSil-Ph) were confirmed by X-ray diffraction (XRD), Fourier transform infrared (FTIR) spectroscopy, and thermogravimetric analysis (TGA). Identical drilling fluid formulations were designed for comparison using MSils and a commercial viscosifier. The rheological properties of fluids were measured at ambient conditions as well as at high temperatures (up to 150 °C) and high pressure (70 MPa). Owing to strong covalent linkages, drilling fluids that were formulated with MSils showed a 19.3% increase in yield point (YP) and a 31% decrease in apparent viscosity (AV) at 150 °C under 70 MPa pressure, as compared to drilling fluids that were formulated with traditional organoclay. The higher yield point and lower apparent viscosity are known to facilitate and increased drilling rate of penetration of the fluids and an enhanced equivalent circulation density (ECD), the dynamic density condition, for efficient oil and gas wells drilling procedures.

## Introduction

The properties of drilling fluids govern the successful completion of oil and gas well drilling operations. It has been well established that the non-productive time (NPT), owing to the deteriorating performance of drilling fluids, has largely amplified the cost of drilling operations and delayed the production of oil and gas from the reservoirs^1^. The principal functions of drilling fluids are^1–4^ (i) suspending and transporting formation cuttings from the bottom of the wellbore to the surface, (ii) suspending formation cuttings during the shutdown of drilling operations, (iii) counter balance the formation pressures to prevent in-flow of gas, oil or water from rocks, (iv) forming a filter cake on the formation surface to improve wellbore stability, and (v) lubricating the drilling tools and drill pipes. There are mainly two types of drilling fluids employed in field operations, oil-based drilling fluids and water-based drilling fluids^5–13^. Water-based drilling fluids (WBMs) are often known for their environmentally benign characteristics, albeit unfavorably high viscosity and lack of stability under high temperature conditions have restricted their applications to certain hydrocarbon reservoirs^7,8^. Oil-based drilling fluids, also known as oil-based muds (OBMs) or as invert emulsion fluids (IEF), have demonstrated wide acceptance in oil and gas drilling operations on account of their stability under extreme rock and reservoir conditions, e.g. high temperature and high pressure^6^. The water-in-oil invert emulsions in OBMs have shown low to moderate viscosity that reduce the energy requirement to pump the fluids and significantly improve the rate of penetration. The key merits of OBMs over water-based drilling fluids are their abilities to perform in soluble salt, water sensitive formations, and offers low frictions^1^.

Drilling fluid formulations are composed of several additives, e.g. oil as a base fluid, an aqueous phase as an internally emulsified phase, viscosifiers, fluid loss additives, rheological modifiers, primary and secondary emulsifiers, wetting agents, pH controller, and weighting agents^1,4,7^. These complex mixtures of additives in fluids address the stability of OBMs under the desired wellbore conditions and provide efficient drilling operations of oil and gas wells. One of the most vital among these additives is the viscosifier, because it preserves the viscosity of the fluids over wide range of temperatures. Numerous viscosifiers have been developed in last five decades and the majority of these viscosifiers are based on organically modified natural layered materials, also known as organoclays^1^.

**Scheme 1:**
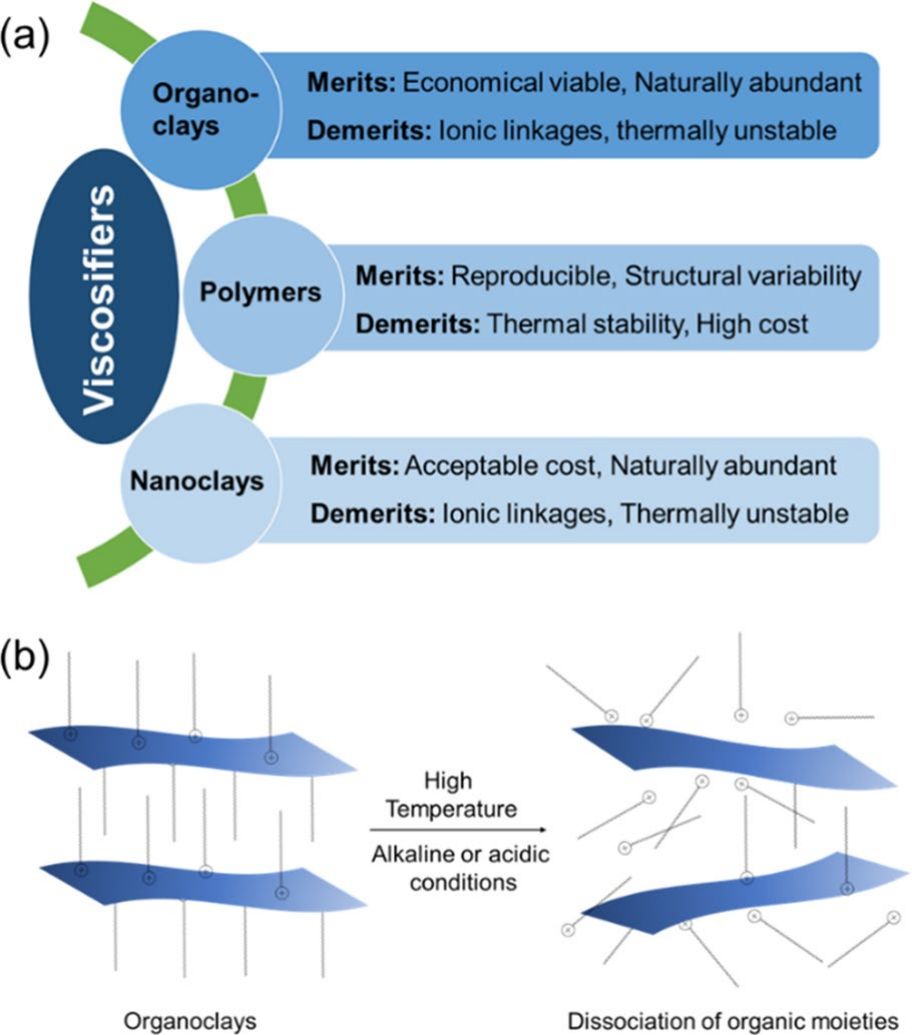
Development of viscosifiers and their shortcomings. (**a**) Chronological development of viscosifiers for oil-based drilling fluids over the last 50 years. (**b**) Effect of the extreme downhole conditions, high temperature, on the traditional organoclays.

The historical developments in the area of various viscosifiers that have been employed as additives in drilling fluids are summarized in Scheme 1a. Organoclays have been employed as a viscosifying additive in drilling fluid formulations since the 1970s. Organoclays are produced through an ion-exchange reaction between cationic clays and quaternary ammonium salts^14^. The resulting organophilic clays can easily be dispersed in an oil- or diesel-based medium that imparts viscosity to the drilling fluids. Since organoclays have been synthesized from naturally abundant clay minerals, they are relatively low cost viscosifiers to manufacture. The refining of crude oil into value-added chemicals and polymers in the early 1980s has allowed for additional development in the area of modified polymers, which can also generate viscosity in the base fluid medium. However, the high cost and thermal degradation of polymers have restricted their wide scale deployment as a fiscally favorable additive in drilling fluid formulations. Researchers in the upstream petroleum sectors established techniques to control the particle size of organoclays to obtain nanoclays in the beginning of twenty-first century. The size reduction of organoclays allow for better dispersion of their nanometer-thick alumino-silicate platelets in the organic phase, however, the electrostatic interaction of organic moieties with layered materials remains as one of the unresolved characteristics. We have developed the next generation of viscosifiers to overcome the disadvantages associated with the current clay-based and polymeric viscosifying additives.

The organic functionalities in organoclays and nanoclays are attached through electrostatic bonding on the surface of layered materials (Scheme 1b). Drilling fluids often experience very high temperatures under downhole conditions, in addition to an alkaline/acidic environment. The organic functionalities are isolated from the layered materials, thereby losing their ability to contribute to the viscosifying properties in drilling fluids. Therefore, it is very important to have strong linkages between the layered materials and organic functionalities to preserve the rheological properties of the drilling fluids.

We have designed and synthesized layered materials that have covalently-linked organic functionalities. Two types of synthetic magnesium silicates (MSils) were prepared, bearing hexadecyl (MSil-C16) or phenyl groups (MSil-Ph) through a facile synthetic route. The synthesis of MSil without organic functionality (MSil-OH) was also demonstrated in order to compare the structural changes upon organic functionalization. The formation of layered structures was evaluated by X-ray diffraction and covalent bonding of organic moieties with nanometer-thick magnesium silicates was revealed by infrared spectroscopic analyses. The thermal stabilities of MSils were studied by thermogravimetric analysis. MSil-C16 and MSil-Ph were incorporated in the drilling fluids to demonstrate the effect of covalently-linked organic moieties. We have also compared the rheological properties of drilling fluids with commercial organoclay under identical conditions to establish the unique characteristics of MSils. The rheological properties were analyzed at high temperatures (up to 150 °C) and high pressures (70 MPa) to simulate the wellbore conditions.

## Materials and methods

### Materials

Magnesium chloride hexahydrate (98%), phenyltrimethoxysilane (97%), hexadecyltrimethoxysilane (95%), tetraethyl orthosilicate (98%), methanol (99.8%), and sodium hydroxide (technical) were received from MilliporeSigma. Commercial organoclay (Claytone HT) was obtained from BYK-CHEMIE GMBH. Commercial drilling fluid additives were obtained from Schlumberger, USA. All chemicals were used as received.

### Characterizations

Powder X-ray diffraction patterns of MSils were recorded by Rigaku benchtop Miniflex 600, equipped with monochromatic X-ray source (600 W) and a D/teX Ultra 1D silicon strip detector. Thermogravimetric analyses (TGA) of MSils were carried out on the SDT q600 TA instrument. The sample was heated up to 800 °C with a 10 °C/min heating rate under a 20 mL/min N_2_ flow rate. Fourier transform infrared (FTIR) sprectra were recorded in attenuated total reflection (ATR) mode within the range 400–4,000 cm^–1^ using a Bruker Tensor 37 FTIR (MiD IR/ATR) spectrometer. Viscoelastic properties of the OBMs were measured using a MCR 303 rheometer from Anton Parr. The storage modulus and loss modulus of the fluids were recorded at different temperatures under 3.45 MPa. The angular frequency was varied between 0.03 and 70 rad/s. The couette coaxial cylinder rotational viscometers (Model 35 Rheometer and iX77 Rheometer, Fann Instrument Company) were used to simulate wellbore conditions for studying the rheological properties of the OBMs. Model 35 Rheometer was employed to obtained rheological properties at ambient conditions while iX77 Rheometer was utilized to record the rheological properties at high temperatures (up to 150 °C) and high pressure (70 MPa). These rheometers offer a true simulation of the most significant flow process conditions encountered during drilling operations. Dial deflection torque readings (600, 300, 200, 100, 6, and 3 rpm) from the rheometers were recorded for the OBMs before and after ageing at 150 °C. Aged OBMs were tested at high temperature and high pressure to obtain data for each OBM. Plastic viscosity (PV), apparent viscosity (AV), and yield point (YP) were calculated from the dial reading recorded on the rheometers: PV = dial reading (600 rpm) – dial reading (300 rpm); YP = dial reading (300 rpm) – PV, AV = dial reading (600 rpm) ÷ 2. Gel strength of the OBMs were measured after holding the OBMs at 10 s and 10 min, followed by applying 3 rpm rotation in the rheometer. The dial readings were recorded and represented as gel strength of the drilling fluids.

### Synthesis of MSil-OH, MSil-C16, and MSil-Ph

Organically modified synthetic magnesium silicates were prepared according to the reported technique with minor modifications^15–17^. Detailed syntheses of MSils are given in Supplementary Information. Briefly, 0.08 mol of silane compound (tetraethyl orthosilicate, hexadecyltrimethoxysilane or phenyltrimethoxysilane) was added to a solution of magnesium chloride hexahydrate (0.06 mol) in 300 mL methanol with stirring at 25 °C. Subsequently, 0.5 M aqueous sodium hydroxide was metered through a peristaltic pump until the pH reached 11. The resulting precipitates were refluxed with stirring at 80 °C for 48 h. The reaction mixtures were cooled to room temperature, followed by filtrations and washing with de-ionized water. The products were dried under vacuum for 24 h and denoted as MSil-OH, MSil-C-16 and MSil-Ph.

### Drilling fluid formulations

Drilling fluids (OBM1, OBM2, and OBM3) were prepared through high shear mixing of the additives (Table 1) in base fluid to form stable oil-in-water emulsions at 11,500 rpm. It is very important to follow the time and order of mixing for each additive to formulate the OBMs. The order of mixing and time of shearing after adding each component is as follows: Step 1 (1–2 min in each step): Diesel (178 g) → organoclay or MSil-C16 or MSil-Ph (2 g) → VersaMul (10 g) → VersaCoat ( 7 g) → Lime (10 g) → Priamine 1074 (3 g) → Shear for 20 min. Step 2 (1–2 min in each step): CaCl_2_ brine (85 g) → VersaTrol HT (4 g) → Shear for 20 min. Step 3: Barite (280 g) → Shear for 20 min. Step 4: RevDust (50 g) → Shear for 5 min. Step 5 (ageing) hot rolled the OBMs at 150 °C under 3.45 MPa in a pressure cell for 16 h.

**Table 1:** Drilling fluid formulations—OBM1, OBM2, and OBM3—with various additives.

Fluid additives	Amount, g	OBM1	OBM2	OBM3
Base fluid – oil phase	178	Diesel	Diesel	Diesel
Viscosifiers	2	Organoclay	MSil-C16	MSil-Ph
Lime	10	Ca(OH)_2_	Ca(OH)_2_	Ca(OH)_2_
Primary emulsifier	10	VersaMul	VersaMul	VersaMul
Secondary emulsifier	7	VersaCoat	VersaCoat	VersaCoat
Rheological modifier	3	Priamine 1074	Priamine 1074	Priamine 1074
Internal aqueous phase	85	CalCl_2_ brine	CalCl_2_ brine	CalCl_2_ brine
Fluid loss additive	4	VersaTrol HT	VersaTrol HT	VersaTrol HT
Weighting material	280	Barite	Barite	Barite

Oil to water phase ratio was 70:30 and the drilling fluid density is 1.61 g/cm^3^. Priamine 1074, Lime, VersaMul, VersaCoat, VersaTrol HT, and barite are commercial trade names of the drilling fluid additives. The density of CaCl_2_ brine is 1.1 g/cm^3^. 50 g of Ca-montmorillonite (RevDust) was added into each OBM to replicate the drilled rock contamination effect that occurs during drilling operations*.*

## Results and discussion

**Scheme 2:**
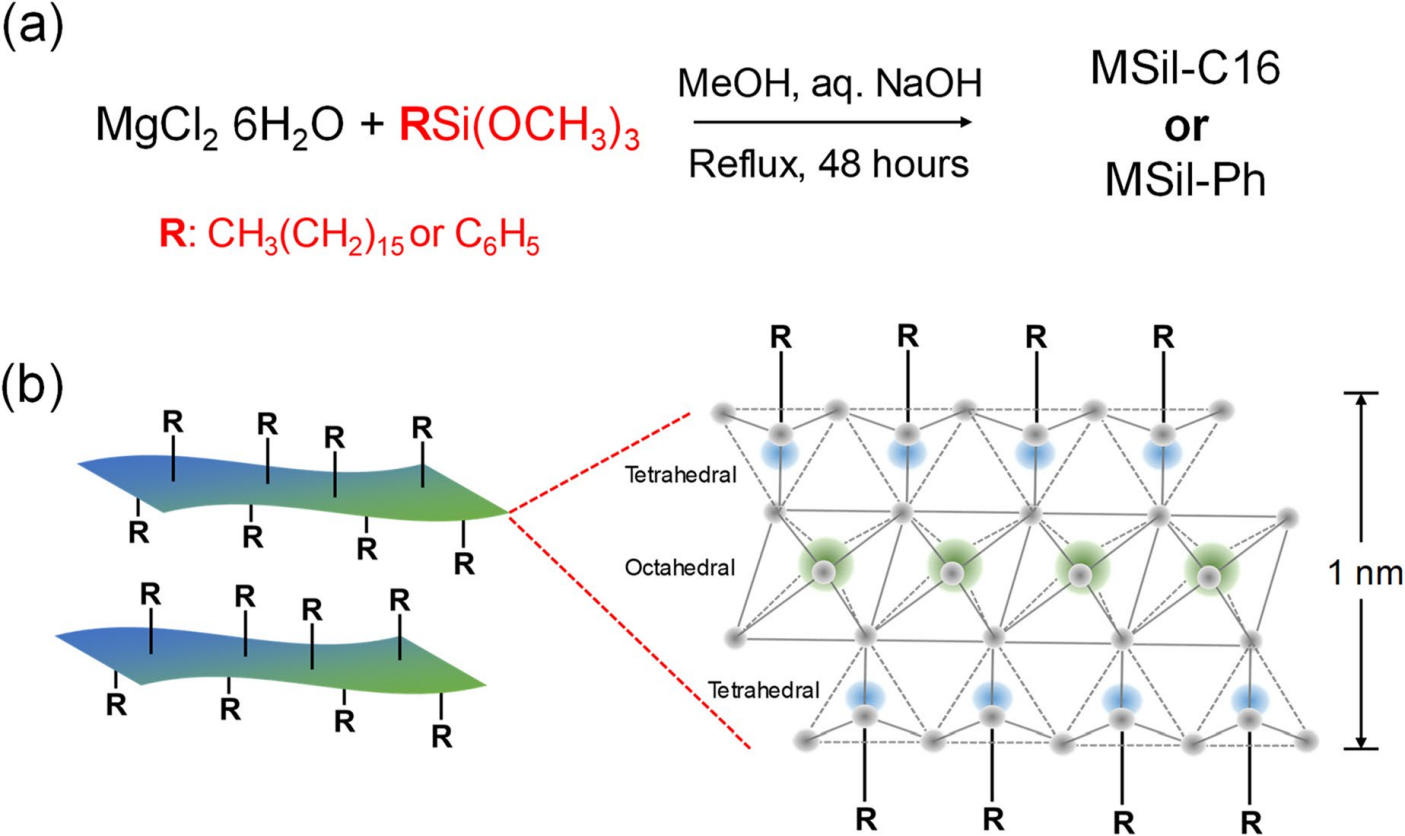
Synthesis and structural characteristics of MSils. (**a**) Reaction route to generate MSil-C16 and MSil-Ph under mild reaction conditions. (**b**) Covalently-linked layered silicate composed of ~ 1 nm thick tetrahedral-octahedral-tetrahedral platelets.

A facile synthetic approach has applied for the preparation of organically modified magnesium silicates (MSils). The architectures of these layered materials were created from the combination of precipitation and sol–gel techniques (Scheme 2). Under alkaline conditions, magnesium salts are precipitated as brucite sheets (octahedral magnesium hydroxide/oxide) and tetrahedral silicates are attached on the brucite sheets during condensation reaction through the sol–gel process^18,19^. The seed crystals of the brucite layers act as structure directing agents and resulting in nanometer-thick magnesium silicate platelets. Pendant groups of organosilanes also facilitate the formation of lamellar structures, due to the hydrophobic nature of the organic functionalities^20^.

**Figure 1:**
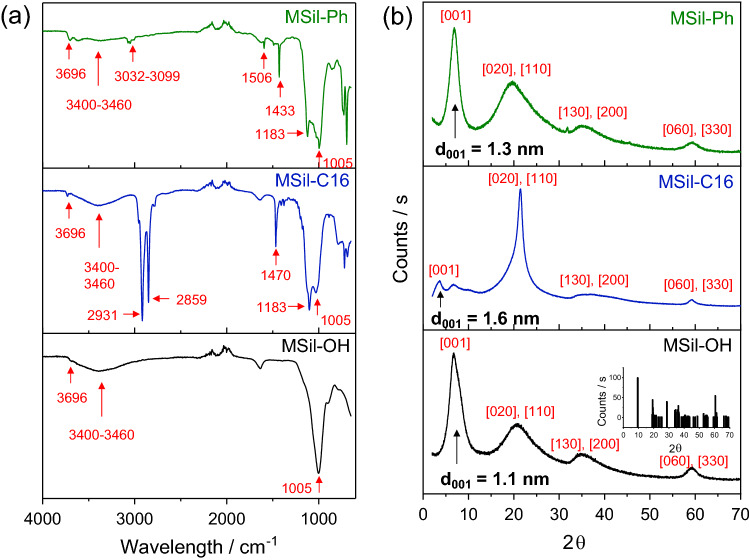
Formation of covalently-linked MSils. (**a**) FT-IR spectrum of MSil-OH, MSil-C16 and MSil-Ph show annotated characteristic stretching vibrations. (**b**) XRD patterns of MSil-OH, MSil-C16 and MSil-Ph (*Inset: ICDD XRD pattern for Si*_*4*_*Mg*_*3*_*O*_*12*_*(OH)*_*2*_*, magnesium silicates hydrates, PDF card # 00–019-0,770*) with crystallographic reflections assignment that show the formation of layered structures.

The formation of covalently-linked magnesium silicates—MSil-OH, MSil-C16, and MSil-Ph—was studied by recording and evaluating Fourier Transform Infrared Spectroscopic (FTIR) spectrum and Powder X-ray diffraction (XRD) patterns. Layered magnesium silicates and organic functional groups show characteristic vibration signals that confirmed the generation of the desired materials (Fig. 1a). The covalent bonding, Si–C, is clearly visible from the stretching at 1,183 cm^–1^ in MSil-C16, and MSil-Ph. Stretching bands at 3,032–3,099 cm^–1^, 1506 cm^–1^, and 1,433 cm^–1^ in MSil-Ph are attributed to C–H aromatic stretch, C–C stretch in the aromatic rings, and Si–H_5_C_6_, respectively. The organic moieties attached with layered materials can be identified from the vibrational band of the alkyl or aromatic functional groups. The vibrational bands^21^^‒^^22^ of aliphatic C–H and aliphatic C–C correspond to 2,931–2,859 cm^–1^ and 1,470 cm^–1^ for MSil-C16. Hydroxyl groups in the magnesium silicates show characteristic broad signals around 3,460–3,400 cm^–1^. Phyllosilicates^23^ (2:1) that are composed of MgO/OH sandwiched between silica tetrahedral provide distinctive stretching bands at 3,696 cm^–1^ and 1,005 cm^–1^ for MgO–H and Si–O–Si, respectively.

Magnesium silicates are the most common class of 2:1 phyllosilicate mineral. In these minerals, Mg in octahedral coordination with O–H that are bound to two sheets of Si in tetrahedral coordination with O atoms. Since one of the O atoms is replaced by organic functionalities in organosilanes, it is expected to form Si tetrahedral with Si–C covalent linkages in layered magnesium silicates. Thus, organic functionalities are located in the interlayer space between layered materials. The formation of 2:1 phyllosilicates structure in these synthetic magnesium silicates was proven through crystallographic reflections in XRD patterns (Fig. 1b). The [001] reflection represents basal spacing or interlayer spacing of magnesium silicates and it proves the information of organic functionalities situated within interlayer space^24,25^. The d_001_ for MSil-OH, MSil-C16, and MSil-Ph is 1.1, 1.6, and 1.3 nm, respectively and these various in interlayer spacing suggest that hexadecyl chains and phenyl groups are located between the inorganic platelets. The diffraction patterns of MSils have also compared with standard magnesium silicates—Si_4_Mg_3_O_12_(OH)_2_—as shown in Fig. 1b (inset). The XRD patterns of standard magnesium silicates were obtained from ICDD (The international Center for Diffraction Data) data base. The diffraction peaks at [020, 110], [130, 200] and [060, 330] are fingerprint reflections of 2:1 phyllosilicate structure. The position of the [060] reflection demonstrates that Mg octahedral sheets are surrounded by three divalent cations and remained unaltered upon introduction of organic groups. It shows that the layered magnesium silicates can accommodate various functionalities without affecting tetrahedral − octahedral − tetrahedral structure^18^.

**Figure 2:**
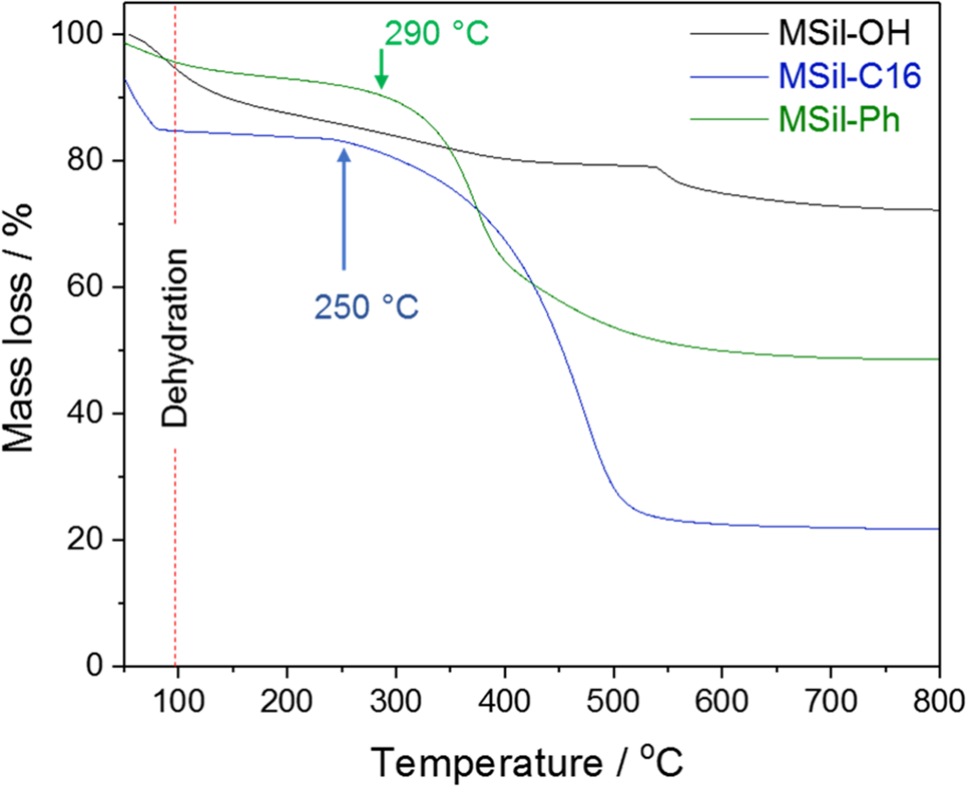
Thermogravimetric analyses of MSil-OH, MSil-C16 and MSil-Ph up to 800 °C.

Drilling fluids have often encountered to extreme temperature and pressure under downhole conditions during oil and gas well drilling operations. Therefore, the additives that are utilized for drilling fluid formulation should show adequate thermal stability under these conditions. We have studied the thermal stability of MSil-OH, MSil-C16 and MSil-Ph up to 800 °C by thermogravimetric analysis (Fig. 2). Initial mass loss in the TGA up to 100 °C corresponds to the removal of adsorbed waters from the magnesium silicates. The degradation of organic moieties in MSil-C16 and MSil-Ph started at 250 and 290 °C, respectively and therefore it is expected that these materials can withstand temperatures within these ranges. The temperature of oil and gas wells generally range from 75–260 °C, and gas wells drilling operations^1^ have encountered temperatures in the range of 150–260 °C. Owing to high thermal stability of MSil-Ph, it can be employed as an additive in drilling fluids in all temperature ranges. Moreover, MSil-Ph has covalently-linked phenyl groups, unlike traditional organoclays that have ionically-linked organic functionalities. MSil-OH shows mass loss (14.7%wt.) within 100–400 °C, which may correspond to tightly bound water molecules.

MSil-OH was synthesized to understand the structural changes upon incorporation of organic functionalities in MSils. MSil-OH is hydrophilic and therefore it cannot be dispersed in organic media. The drilling fluid formulations and their rheological properties studies only focuson the effect of two organophilic MSils. We have employed MSil-C16 and MSil-Ph as viscosifiers in OBMs and they were compared with a commercial viscosifier, organoclay. The OBMs are pumped through drilling strings (drilling pipes), carry cuttings, and return to the ground surface after passing between drill pipes and rock formation (Supplementary Information, Scheme S1). This type of fluid flow process requires measurement in a rheometer that is equipped with coaxial rotational cylinders (Couette type) and thus can provide true simulations of the flow characteristics of OBMs during drilling operations. Rheology of OBMs—OBM1, OBM2, and OBM3—were obtained at ambient conditions (Table 2).

**Table 2:** Dial readings, gel strength at 10 s. and 10 min., plastic viscosity, apparent viscosity, and yield point for OBMs at 25 °C and ambient pressure after ageing at 150 °C for 16 h.

Shear rate	Dial readings		
	OBM1	OBM2	OBM3
600 rpm	63.8	69.1	71.2
300 rpm	43.2	44.5	45.6
200 rpm	33.8	34.1	34.8
100 rpm	26.3	27.8	28.5
6 rpm	14	13.8	14.2
3 rpm	12.3	11.9	12.4
Rheological properties			
Gel strength 10 s., lb/100 ft^2^	13	12.5	13.2
Gel strength 10 min., lb/100 ft^2^	15.2	14.8	15.6
PV, cP	20.6	24.6	25.6
AV, cP	31.9	34.55	35.6
YP, lb/100 ft^2^	22.6	19.9	20

OBMs were aged at 150 °C in a pressure vessel under 3.45 MPa before measuring rheological properties. All the OBMs show shear thinning behavior as a function of shear rates. OBMs should have higher gel strength to suspend formation cuttings. OBM2 and OBM3 show equivalent or better gel strength compared to OBM1 at ambient conditions. This is attributed to excellent dispersion of organophilic layered materials that enhance the viscosity of the organic phase of the drilling fluids. Other rheological properties, such as PV, AV, and YP are also almost similar in all OBMs. This identical rheological property in all OBMs was expected because organoclay has been proven to be stable at low temperature. However, the changes in rheological properties in OBM1 that contains organoclay is clearly visible under higher temperature and high pressure conditions.

**Figure 3:**
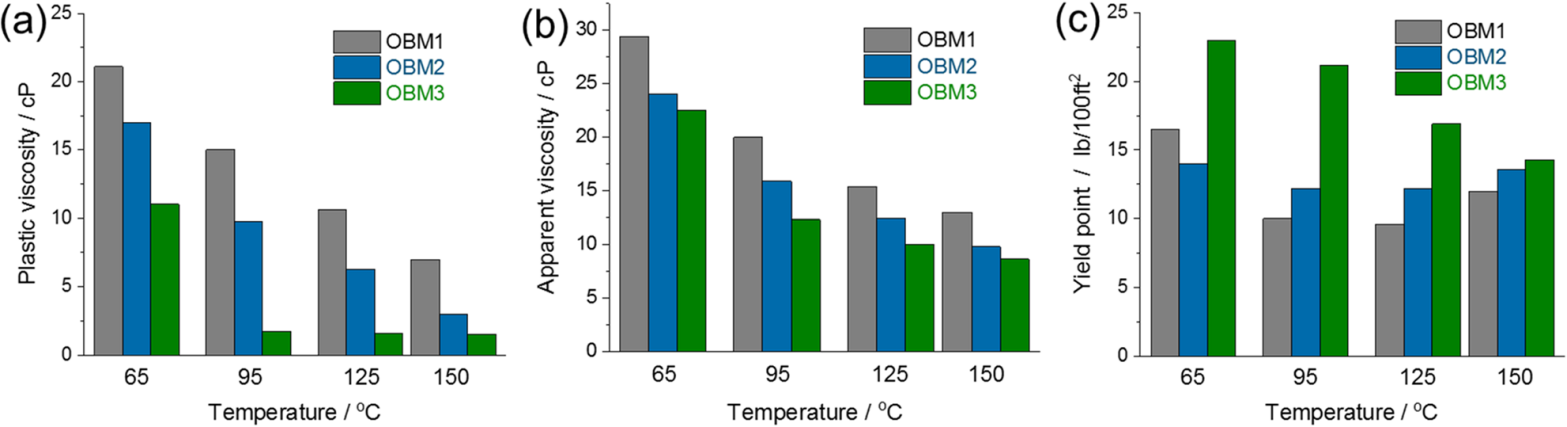
Rheological properties of OBMs. (**a**) Plastic viscosity, (**b**) Apparent viscosity, and (**c**) Yield point, up to 150 °C at 70 MPa.

The fluid flow properties^26–33^—PV, AV, and YP—of the OBMs were derived from the slope of shear stress and shear rate based on the Bingham plastic model. The PV and AV of OBMs provide information on resistance of fluid flow. The resistance to initial fluid flow or stress needed to displace the fluid is known as YP. These properties were determined from the OBMs at different temperatures (65, 95, 125, and 150 °C) under 70 MPa pressure in the rheometer. OBM2 and OBM3 have demonstrated lower PV and AV as compared to OBM1 (Fig. 3a, 3b). Owing to the lower PV and AV in OBM2 and OBM3, they can produce excellent rates of penetration during drilling operations.

The PV of OBM1, OBM2, and OBM3 was observed to be 7, 3, and 1.5 cP at 150 °C under 70 MPa. Likewise, the AV of OBM1, OBM2, and OBM3 was observed to be 13, 9.8, and 8.9 cP at 150 °C under 70 MPa. We have noticed about a 78% decrease in PV and a 31% decrease in AV for OBM3 when compared to OBM1. This phenomenon can be attributed to the smaller size of the silicate platelets of MSils with respect to organoclay. Furthermore, the density of organic functionality is higher in MSil since each silicon atom is attached with an organic moiety. There have been several techniques employed to measure the stability of invert emulsion fluids. In this study, electrical stability tests were conducted to check the invert emulsion stabilities in OBMs (Supplementary Information, Figure S1). The electrical stability of OBM1, OBM2, and OBM3 is 569, 500, and 546 V, respectively. It is well established that electrical stability above 300 V for OBMs is considered stable for invert emulsions.

The interaction between particles and the forces between them are known to contribute largely toward the YP of OBMs. Thus, the thermal stability of YP over wide temperature ranges plays a key role in OBMs. OBMs should have a higher and stable YP under downhole conditions to suspend a large quantity of weighing materials (e.g. barite) and to transport cuttings of drilled rocks during drilling operations. The reduction in YP of OBMs at high temperature causes serious consequences, e.g. stuck pipes that put a halt on drilling operations and increases non-productive time. OBM2 and OBM3 have shown exceptional stability of YP at different temperatures. The YP of OBM1, OBM2, and OBM3 is 12, 13.6, and 14.3 lb/100 ft^2^, respectively at 150 °C (Fig. 3c), which is a 19.2% (OBM3) improvement compared to OBM1. It is clear from the YP of OBMs at varied temperatures that OBM3 has outperformed OBM2 and OBM3. Low shear yield point (LSYP) of OBM3 has exceeded OBM2 and OBM3 within the studied temperature ranges (Supplementary Information, Figure S2).

**Scheme 3:**
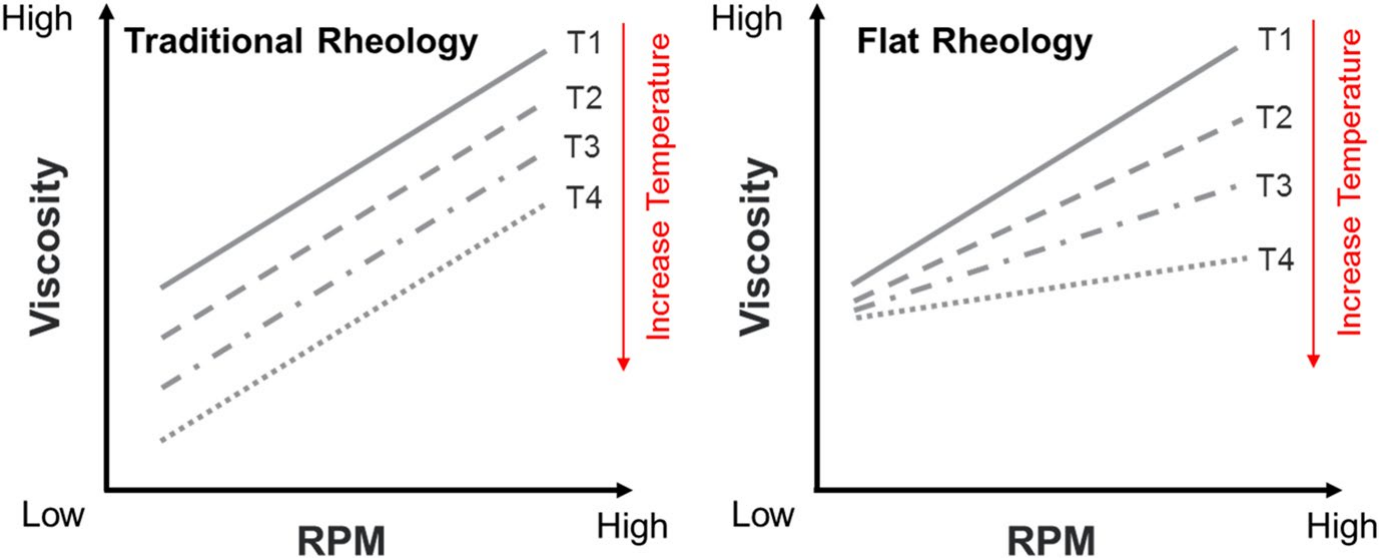
Changes in the rheological properties of drilling fluids with respect to temperature – Traditional rheology *vs* Flat rheology.

The low thermal stability of viscosifiers affects the viscosity of OBMs upon deviation in wellbore temperatures at different depths of the oil and gas wells. A drastic decrease in viscosity may result in poor hole cleaning, solid particles sagging, and disruption in fluid circulation. These difficulties are avoidable, if the OBMs show minimal changes in viscosity over a wide range of wellbore temperatures. A comparison between traditional rheology and flat rheology^28–30,32^ has been illustrated in Scheme 3. In traditional rheology^33^, the viscosities measured at different shear rates decreases with an increase in temperature. The viscosity readings at low rpm have also decreased at higher temperatures. There is an almost linear correlation between reductions in viscosity with increases in wellbore temperature. Nonetheless, if the viscosity readings at low rpm remained unchanged with increases in temperature and dial readings at higher rpm decreased at high temperature, the curves start to become flattened. This minimal sensitivity in flow properties over a range of temperatures has been known within the industry as flat rheology. Flat rheology of the OBMs improves the high temperature rate of penetration and provides better equivalent circulation density during oil and gas well drilling processes.

**Figure 4:**
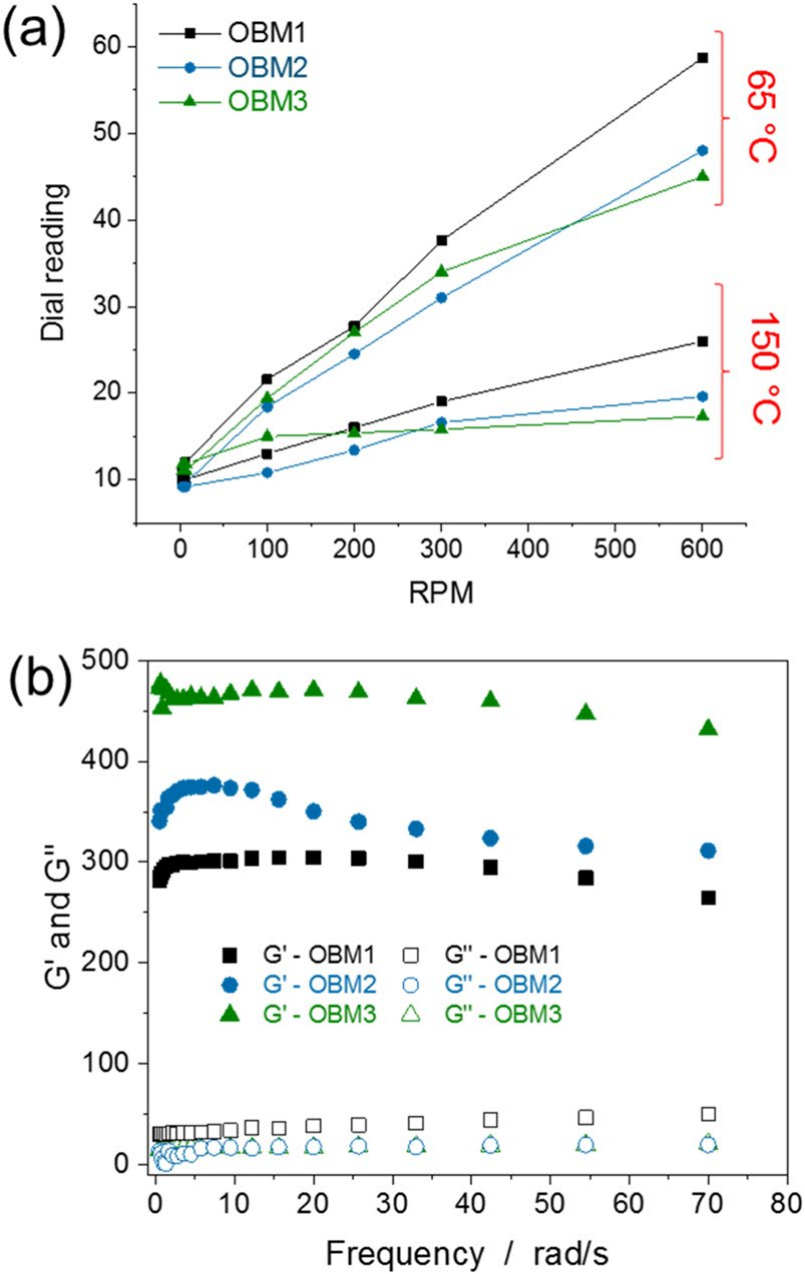
Observation of flat rheology and viscoelastic properties. (**a**) Rheological properties of OBM1, OBM2, and OBM3 under 70 MPa reveal flat rheological behavior for OBM3 with increase in temperature. (**b**) Storage modulus and loss modulus of OBM1, OBM2, and OBM3 under 3.45 MPa at 150 °C.

Interestingly, OBM3 shows flat rheological performance where it revealed minimal changes in low shear viscosity upon increases in temperature (Fig. 4a, Supplementary Information Figure S3). The dial readings at 3 rpm and 600 rpm are observed to be 11.8 cP and 17.3 cP for OBM3, whereas, dial readings of OBM1 at 3 rpm and 600 rpm are 10 cP and 26 cP, at 150 °C and 70 MPa. The order of flat rheology of the OBMs that have been studied in this research is OBM1 < OBM2 < OBM3. This unprecedented property of flat rheology in OBM1 and OBM2 could be ascribed to covalently-linked organic moieties on magnesium silicates, MSil-C16 and MSil-Ph. These two MSils have hexadecyl and phenyl functionalities that are linked through Si–C linkages. We believe that these strong chemical linkages in MSils have not allowed organic functionalities to be detached from the inorganic platelets. Ionic or electrostatic interactions between organic functionalities and the layered material in organoclay have dissociated under extreme conditions, which resulted in the changes in viscosity of drilling fluids at high temperatures.

The measurement of viscoelastic properties (storage modulus and loss modulus) were carried out to understand the thixotropic behaviors of OBMs at 150 °C under a confined pressure of 3.45 MPa (Fig. 4b). The strength of the gel formations improves suspension of high density materials and allow excellent suspension of drill cuttings, as we have already discussed in detail in this high temperature high pressure study of OBMs. Storage modulus (G’) and loss modulus (G’’) are represented as typical physical characteristics, e.g. gel-like and fluid-like behaviors. Higher G’ over G’’ at a given frequency reveals that the elastic property or gel strength of OBMs and fluid-like behavior is dominated if G’ is lower than G’’. The G’ has remained higher than G’’ for OBM1, OBM2, and OBM3 at all frequencies, suggesting that all OBMs have a gelation state. OBM1 demonstrated highest G’ when compared to OBM2 and OBM3 as a result of the excellent dispersion of MSil-Ph in invert emulsion fluids. High density phenyl functionalities on magnesium silicates allow formation of a colloidal dispersion in organic media. Traditional organoclay undergoes structural dissociation in OBM1 at high temperatures. The rigidity of phenyl functionality over hexadecyl functionality in MSils is responsible for the greater gel-like behavior in OBM1 compared to OBM2. It is significant to note that the synthetic nature of MSils, with minimum impurities and covalently-linked organic functionalities, contribute to the enriched rheological properties in OBMs compared to organoclay.

## Conclusions

The application of MSils, comprised of covalently-linked organic functionalities on the nanometer-thick layered material, in reservoir drilling fluids have been successfully proven under extreme wellbore conditions. Organoclays have lost their functions as viscosifying fluids at high temperature because of their weak ionic linkages of organic moieties with alumino-silicates. As a result of facile synthetic routes, MSils have been prepared with minimum impurities and desired organic functionalities that were linked through Si–C bond. XRD patterns of MSil-OH, MSil-C16, and MSil-Ph have shown formation of 2:1 phyllosilicate structure and it has also revealed the site of organic functionalities within the interlayer spaces of layered materials, supported by an increase in the d_001_ basal spacing. FT-IR spectrum showed characteristic vibration bands for structural features of MSils proving the formation of organically linked layered magnesium silicates. MSil-C16 and MSil-Ph displayed thermal stability of 250 °C and 290 °C, confirmed by TGA. Drilling fluid formulations—OBM1, OBM2, and OBM3—were prepared using various additives to obtain stable invert emulsion fluids. Rheological measurements of thermally aged OBMs were carried out to determined PV, AV, and YP of OBMs at different temperatures under 70 MPa. OBM3 has the lowest PV (1.5 cP) compared to OBM1 (7 cP) and OBM2 (3 cP) at 150 °C, which is expected to deliver excellent rate of penetration. Simultaneously, the YP of OBM3 was 14.3 lb/100 ft^2^, which is higher than OBM1 and OBM2 at high temperatures, a property that provides rock cutting carrying capacity. In addition to better PV, AV, and YP of OBMs that contained MSils, we have noticed minimal changes in viscosities of the fluids with an increase in temperature. OBM3 has exhibited flat rheological behaviors due to the presence of MSil-Ph in the formulations. The improved rheological properties in OBM2 and OBM3 correspond to the novel materials chemistries of MSils―synthetic nanometer-thick platelets and covalently-linked organic functionalities.

## Supplementary information

Supplementary information.
