# Supplementary material for: Organically modified layered magnesium silicates to improve rheology of reservoir drilling fluids
Source: Sci Rep. 2020 Aug 17;10:13851. doi: 10.1038/s41598-020-70752-1 (PMC7431547; doi:10.1038/s41598-020-70752-1)
Supplement: Supplementary file 1 — Supplementary information. [file 41598_2020_70752_MOESM1_ESM.pdf]

Supplementary Information

Organically Modified Layered Magnesium Silicates to Improve Rheology of Reservoir Drilling Fluids

Hasmukh A. Patel* and Ashok Santra

Drilling Technology Team, Aramco Americas: Aramco Research Center – Houston

16300 Park Row Dr, Houston, TX 77084, United States

*E-mail: hasmukh.patel@aramcoamericas.com

Materials

Drilling fluid additives – VersaMul, VersaCoat, and VersaTrol – are obtained Schlumberger, USA. Priamine 1074 was received from Croda Coating and Polymers, USA. Calcium hydroxide (commercial grade), Magnesium chloride hexahydrate (98%), phenyltrimethoxysilane (97%), hexadecyltrimethoxysilane (95%), tetraethyl orthosilicate (98%), methanol (99.8%), and sodium hydroxide (technical) were received from MilliporeSigma. Commercial organoclay (Claytone HT) was obtained from BYK-CHEMIE GMBH. All chemicals were used as received without any further purification.

Syntheses of Synthetic Magnesium Silicates (MSils)

MSil-OH

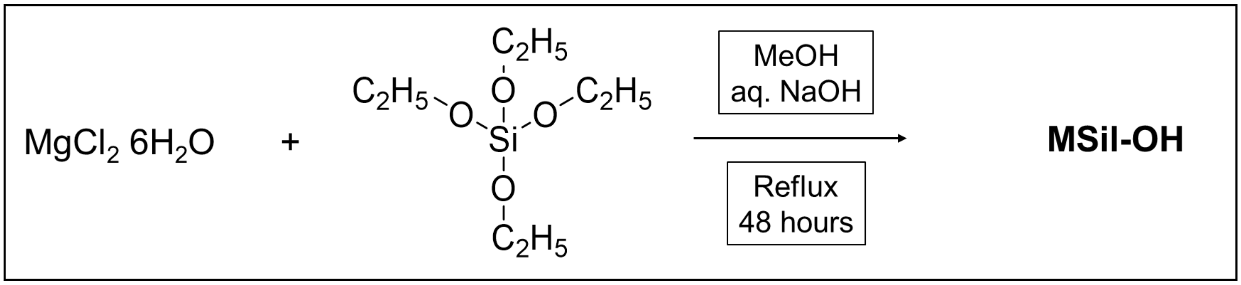

Magnesium chloride hexahydrate (12.2 g, 0.06 mmol) was dissolved in 300 mL of methanol. Tetraethyl orthosilicate (16. 7 g, 0.08 mol) was added dropwise into the solution. Under vigorous stirring, aqueous sodium hydroxide solution (0.5 M) was slowly added through peristaltic pump until the pH reached 11 at 25 °C. The white suspension was allowed to reflux at 80 °C for 48 h with continuous stirring. The resulting white precipitates were filtered and washed with de-ionized water thrice (25 mL X 3). The product was dried at 80 °C under vacuum for 24 hours and it is denoted as MSil-OH.

MSil-C16

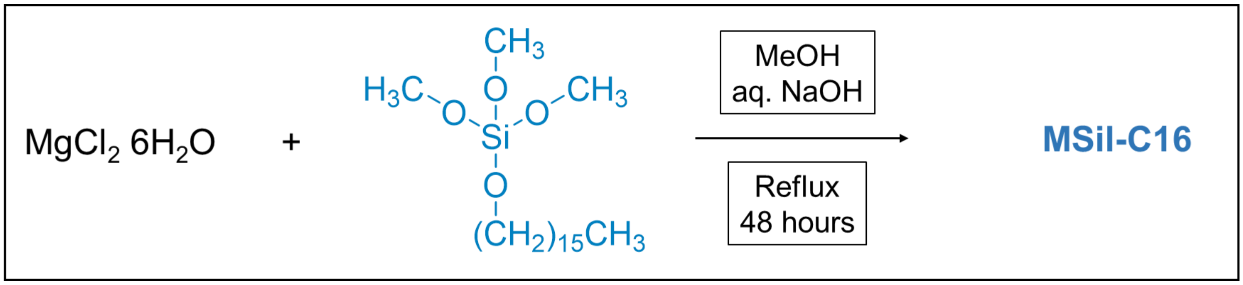

Magnesium chloride hexahydrate (12.2 g, 0.06 mmol) was dissolved in 300 mL of methanol. Hexadecyltrimethoxysilane (27.7 g, 0.08 mol) was added dropwise into the solution. Under vigorous stirring, aqueous sodium hydroxide solution (0.5 M) was slowly added through peristaltic pump until the pH reached 11 at 25 °C. The white suspension was allowed to reflux at 80 °C for 48 h with continuous stirring. The resulting product was filtered and washed with de-ionized water thrice (25 mL X 3). White wax-like product was dried at 25 °C under vacuum for 24 hours and it is denoted as MSil-C16.

MSil-Ph

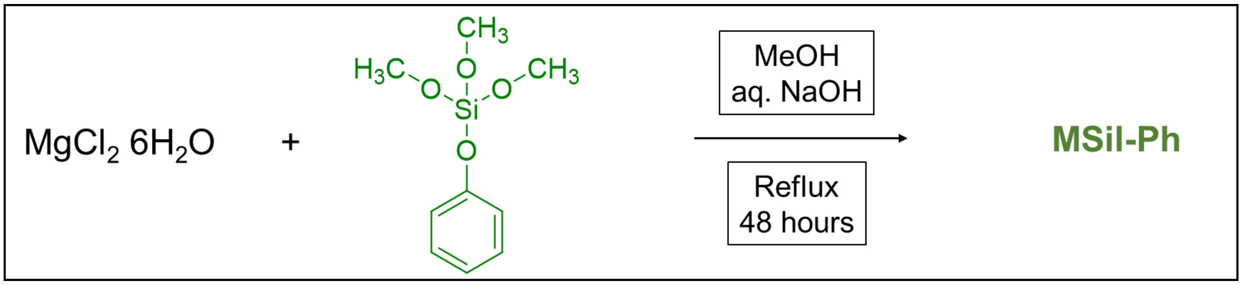

Magnesium chloride hexahydrate (12.2 g, 0.06 mmol) was dissolved in 300 mL of methanol. Phenyltrimethoxysilane (15. 9 g, 0.08 mol) was added dropwise into the solution. Under vigorous stirring, aqueous sodium hydroxide solution (0.5 M) was slowly added through peristaltic pump until the pH reached 11 at 25 °C. The white suspension was allowed to reflux at 80 °C for 48 h with continuous stirring. The resulting white precipitates were filtered and washed with de-ionized water thrice (25 mL X 3). The product was dried at 80 °C under vacuum for 24 hours and it is denoted as MSil-Ph.

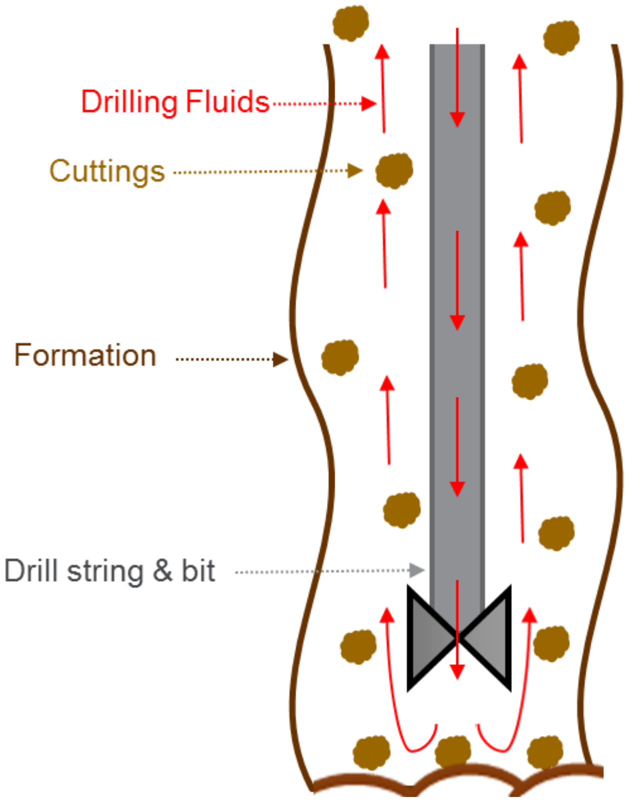

Scheme S1. Typical drilling operation diagram. Drilling fluids are pumped through drilling string & bit. Drilling fluids carried the formation cuttings through the gap between drill pipe and formation to the ground surface.

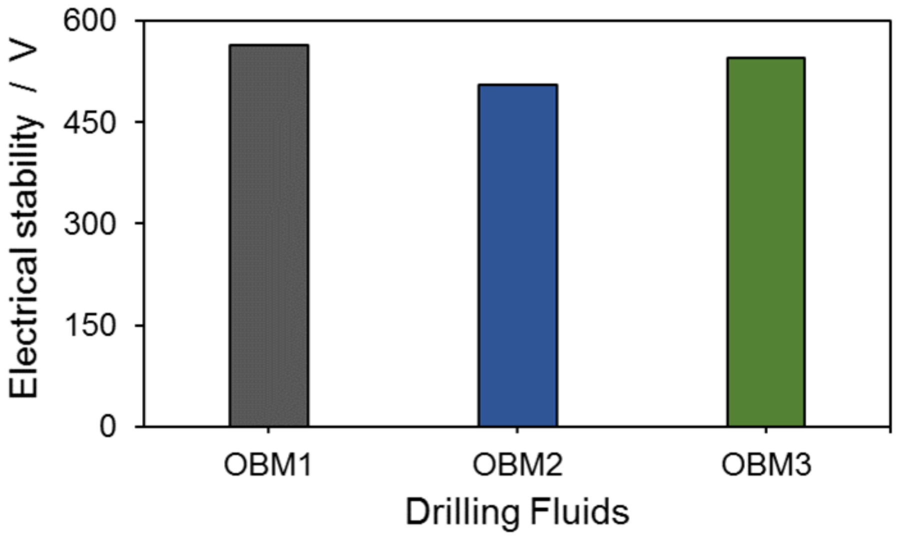

Figure S1. Invert emulsion stability of the OBM1, OBM2, and OBM3. All OBMs were aged at 150 °C for 16 hours before electrical stability tests.

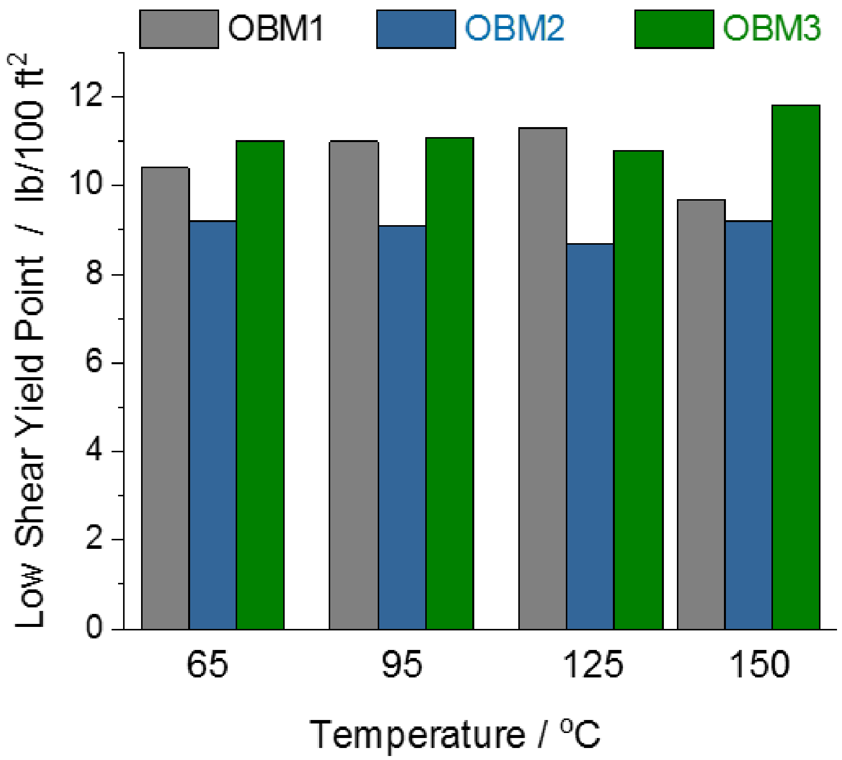

Figure S2. Low shear yield point for OBM1, OBM2, and OBM3 at different temperatures (65, 95, 125, and 150 °C) under 70 MPa of pressure.

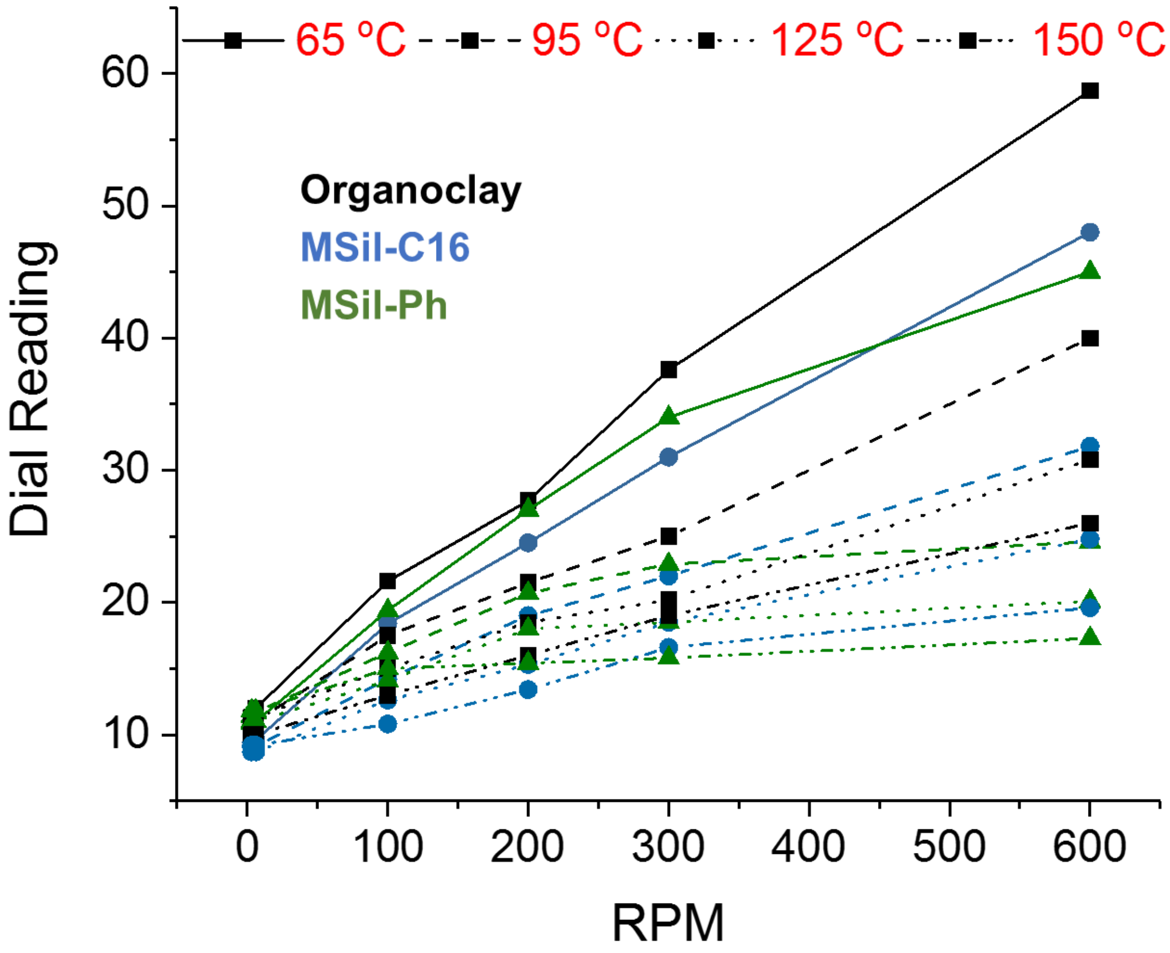

Figure S3. Rheological properties of OBM1, OBM2 and OBM3 under 70 MPa reveal flat rheological behavior for OBM3 with increase in temperature. *Solid line: 65 °C, dash line: 95 °C, Solid Dash line: 125 °C, Solid Dash Dash line: 150 °C.*
